# Charge‐Transfer‐Controlled Growth of Organic Semiconductor Crystals on Graphene

**DOI:** 10.1002/advs.201902315

**Published:** 2020-02-14

**Authors:** Nguyen Ngan Nguyen, Hyo Chan Lee, Min Seok Yoo, Eunho Lee, Hansol Lee, Seon Baek Lee, Kilwon Cho

**Affiliations:** ^1^ Department of Chemical Engineering Pohang University of Science and Technology Pohang 37673 Republic of Korea

**Keywords:** charge transfer, graphene, growth template, organic electronics, organic semiconductors

## Abstract

Controlling the growth behavior of organic semiconductors (OSCs) is essential because it determines their optoelectronic properties. In order to accomplish this, graphene templates with electronic‐state tunability are used to affect the growth of OSCs by controlling the van der Waals interaction between OSC ad‐molecules and graphene. However, in many graphene‐molecule systems, the charge transfer between an ad‐molecule and a graphene template causes another important interaction. This charge‐transfer‐induced interaction is never considered in the growth scheme of OSCs. Here, the effects of charge transfer on the formation of graphene–OSC heterostructures are investigated, using fullerene (C_60_) as a model compound. By in situ electrical doping of a graphene template to suppress the charge transfer between C_60_ ad‐molecules and graphene, the layer‐by‐layer growth of a C_60_ film on graphene can be achieved. Under this condition, the graphene–C_60_ interface is free of Fermi‐level pinning; thus, barristors fabricated on the graphene–C_60_ interface show a nearly ideal Schottky–Mott limit with efficient modulation of the charge‐injection barrier. Moreover, the optimized C_60_ film exhibits a high field‐effect electron mobility of 2.5 cm^2^ V^−1^ s^−1^. These results provide an efficient route to engineering highly efficient optoelectronic graphene–OSC hybrid material applications.

## Graphical abstract

The growth behavior of fullerene (C_60_) thin films on graphene templates where charge transfer occurs is presented. The number of electrons transferred from graphene to C_60_ is controlled by in situ electrical gating of graphene during C_60_ deposition. When this electron transfer is suppressed, high‐crystalline C_60_ thin films are achieved for highly efficient optoelectronic applications.

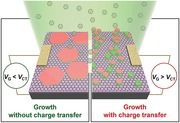

## 1 Introduction

Graphene has excellent properties, so the possibility of integrating it with both inorganic and organic semiconductors has been intensively studied. Graphene–semiconductor heterostructures provide multifunctionality and desirable properties for scalable and flexible optoelectronic applications.^1^, ^2^ The ideally sp^2^‐hybridized carbon atoms of graphene constitute a basal plane with no dangling bonds, so it provides an atomically clean interface with a semiconductor; this contact is extraordinary and cannot be achieved with traditional interfaces. With the introduction of these unique graphene–semiconductor interfaces, researchers have proposed various graphene–semiconductor hybrid optoelectronic devices such as field‐effect transistors (FETs), light‐emitting diodes, solar cells, photodetectors, and barristors.^3^, ^4^, ^5^

Graphene is inert and is composed of a single‐atom‐thick layer, so it is a useful growth template for semiconductors, especially organic semiconductors (OSCs).^6^, ^7^ The assembly of OSC thin films on graphene is mainly determined by the interactions between OSC ad‐molecules and the graphene template (e.g., van der Waals). Therefore, the graphene template can enable epitaxial growth of highly crystalline OSC thin films.^8^ In addition, these interactions can easily be tuned by controlling the electronic properties of graphene,^9^, ^10^ so graphene templates offer a facile and direct approach to prepare graphene–OSC heterostructures with desirable interfacial properties. However, despite the great potential of graphene–OSC heterostructures, only a few studies of OSCs' growth behavior on electronic‐states‐controlled graphene have been reported.^7^, ^10^ Therefore, to develop a reliable method to optimize the growth of OSCs on graphene templates, the complex of OSC molecules and graphene templates and possible interactions between them should be investigated.

Here, we demonstrate that an epitaxial growth of a vacuum‐deposited fullerene (C_60_) thin film on a graphene template can be controlled by tuning charge transfer between them. The Fermi level (*E*_F_) of the graphene template determines the amount of charge transfer between the graphene and the C_60_ ad‐molecules, and this amount in turn affects the molecular dynamics of C_60_ on the graphene template. By finely tuning the *E*_F_ of the graphene template, we induced layer‐by‐layer growth of highly ordered C_60_ films on graphene. Considering that the thin film's topological and crystalline features determine the optoelectronic properties of OSCs,^11^ this approach advances the efficiency of organic electronic devices. The C_60_ films grown under optimized conditions exhibited a maximum field‐effect mobility of 2.5 cm^2^ V^−1^ s^−1^. Furthermore, a graphene–C_60_ Schottky junction prepared by our method approached the Schottky–Mott limit, which is desirable for highly efficient graphene–OSC barristors and other vertical graphene–OSC hybrid optoelectronic devices.

## 2 Results and Discussion

### 2.1 Charge Transfer between Graphene and C_60_

**Figure 1:**
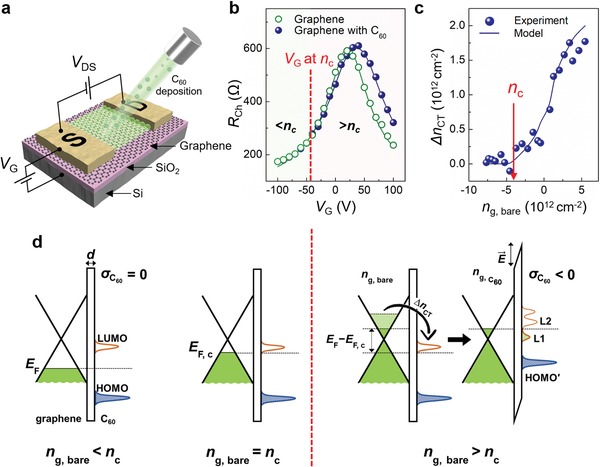
Charge transfer between graphene and C_60_. a) Schematic diagram showing G‐FET with deposited C_60_. b) Transfer characteristic of G‐FET before (green open circle) and after C_60_ deposition (blue closed circle). Solid lines are model fits. c) Concentration of transferred charge carrier after C_60_ deposition Δ*n*_CT_ versus initial charge carrier concentration of bare graphene *n*_g,bare_. d) Energy band diagrams of graphene/C_60_ when *n*_g,bare_ < *n*_c_ (left), *n*_g,bare_ = *n*_c_ (middle), and *n*_g,bare_ > *n*_c_ (right).

We first investigated the transfer of electrons from graphene to C_60_. Analyses using ultraviolet photoelectron spectroscopy, Kelvin probe force microscopy, and Raman spectroscopy revealed that the adsorption of C_60_ molecules induced p‐type doping of graphene (Figure S2, Supporting Information). To clarify the relationship between charge transfer and the initial electronic states of graphene, we fabricated graphene field‐effect transistors (G‐FETs) on 300 nm thick SiO_2_/Si substrates and compared the transfer characteristics of the G‐FETs before and after 3 s of C_60_ deposition at a deposition rate of 5 × 10^−2^ monolayer per second (ML s^−1^) (**Figure** **1**a). To eliminate the contact resistance, we used transfer‐length‐method measurements so that the change in graphene channel resistance (*R*_Ch_) could be solely attributed to the change in charge‐carrier density (*n*_g_, *n*_g_ > 0 for electrons and *n*_g_ < 0 for holes).

After C_60_ deposition, the *R*_Ch_ was preserved as long as the gate voltage (*V*_G_) was <−40 V. This preservation demonstrates that deposition of C_60_ did not cause degradation of graphene, and more importantly, that no charge transfer occurred between graphene and C_60_ in this range of *V*_G_. However, at *V*_G_ > −40 V, the *R*_Ch_ *–V*_G_ curve shifted to the right; this change indicates that electrons were transferred from graphene to C_60_ (Figure 1b). This shift of *R*_Ch_ *–V*_G_ curves when the magnitude of *V*_G_ is larger than a certain value was consistently observed with other samples from different batches (Figure S3, Supporting Information).

To calculate the number of transferred electrons (Δ*n*_CT_ (cm^−2^)) at a certain *V*_G_, the *R*_Ch_ *–V*_G_ curve was fitted using the constant‐mobility model.^12^ Then the carrier density of bare graphene before C_60_ deposition (*n*_g,bare_) and the carrier density of graphene–C_60_ after C_60_ deposition ($n_{\text{g},{\text{C}}_{\text{60}}}$) were each calculated at each *V*_G_ as

(1) $$n_{\text{g}}=\text{sgn}\left(V_{\text{G}}-V_{\text{D}}\right)\sqrt{{\left(\frac{\text{1}}{\mu eR_{\text{Ch}}}\frac{L}{W}\right)}^{\text{2}}-n_{\text{res}}^{2}}$$

where *V*_D_ is *V*_G_ at maximum *R*_Ch_, μ is the carrier mobility, *e* is the elementary charge, *L* is the channel length, *W* is the channel width, and *n*_res_ is the residual carrier concentration in graphene. Then Δ*n*_CT_ was calculated as $n_{\text{g},\text{bare}}-n_{\text{g},{\text{C}}_{\text{60}}}$. Before C_60_ deposition, the fitted values of μ and *n*_res_ of the graphene transistor were 4470 cm^2^ V^−1^ s^−1^ and 2.3 × 10^12^ cm^−2^, respectively. When plotted versus *n*_g,bare_ (Figure 1c), extracted Δ*n*_CT_ showed no charge transfer between graphene and C_60_ when *n*_g,bare_ was less than a critical value, *n*_c_ = −4.4 × 10^12^ cm^−2^. As *n*_g,bare_ approached *n*_c_, charge transfer started and gradually increased with increasing *n*_g,bare_. The *V*_G_‐dependent contact resistance in G‐FETs also supports our claim that the charge transfer occurred when *n*_g,bare_ > *n*_c_ (Figure S4, Supporting Information).

The observed *n*_g,bare_‐dependent charge transfer between graphene and C_60_ is explained as follows. The electrons in graphene are transferred to C_60_ when the *E*_F_ of graphene is higher than the lowest unoccupied molecular orbital (LUMO) level of adjacent C_60_. The LUMO level of isolated C_60_ molecules is known to be −4.5 eV,^13^ which is similar to the *E*_F_ of undoped graphene. However, the energy levels of organic molecules change and broaden upon adsorption of C_60_, because of the polarizability of the substrate;^14^, ^15^ thus, the LUMO level of C_60_ adsorbates can lie below the *E*_F_ of undoped graphene that has *n*_g,bare_ > *n*_c_; as a result, the graphene becomes p‐type doped. The absence of charge transfer when *n*_g,bare_ < *n*_c_ is attributed to the *E*_F_ of graphene being lower than the LUMO level of the C_60_ adsorbates (Figure 1d, left). As the *E*_F_ of graphene is raised by external gating such that it reaches the LUMO level of C_60_, electrons are transferred from graphene to C_60_, and the *E*_F_ of graphene is pinned to the LUMO level of C_60_. As a result of this charge transfer, an electric field is generated between the graphene and the C_60_, so the vacuum level at the interface becomes tilted so that the *E*_F_ of the graphene and the LUMO level of the C_60_ are aligned (Figure 1d, right).

First, the number of charges is conserved at the graphene–C_60_ interface as

(2) $$\frac{C_{\text{g}}}{e}\left(V_{\text{G}}-V_{\text{D},\text{bare}}\right)=n_{\text{g},\text{bare}}=n_{\text{g},{\text{C}}_{60}}+\frac{\sigma_{{\text{C}}_{60}}}{e}$$

where *C*_g_ is the dielectric capacitance, *V*_D,bare_ is the *V*_D_ of the G‐FET before C_60_ deposition, and $\sigma_{{\text{C}}_{\text{60}}}$ is the surface charge density in a C_60_ film. The charge redistribution at the graphene–C_60_ interface as a function of *n*_g,bare_ can be estimated by solving

(3) $$\text{sgn}\left(n_{\text{g},{\text{C}}_{60}}\right)\hbar v_{\text{F}}\sqrt{\pi |n_{\text{g},{\text{C}}_{60}}|}-\text{sgn}\left(n_{\text{c}}\right)\hbar v_{\text{F}}\sqrt{\pi |n_{\text{c}}|}=e\frac{\sigma_{{\text{C}}_{60}}}{\varepsilon_{0}}d$$

where ℏ is the reduced Planck's constant, *v*_F_ is the Fermi velocity of graphene, ε_0_ is the vacuum permittivity, and *d* is a fitting parameter that describes the spacing between graphene and C_60_. The left‐hand side of Equation (3) is *E*_F_ − *E*_F,c_ (Figure 1d), in which *E*_F,c_ is the critical Fermi level where the charge transfer between graphene and C_60_ occurs. The right‐hand side is the charge‐transfer‐induced shift of the vacuum level at the interface.

The *R*_Ch_–*V*_G_ curves of G‐FETs and the Δ*n*_CT_ as a function of *n*_g,bare_ were modelled using calculated $n_{\text{g},{\text{C}}_{\text{60}}}$ and *d*. The models successfully replicated the experimental values (Figure 1b,c). Moreover, the charge transfer modifies the density of states of C_60_ so that the LUMO level of charged C_60_ molecules is split into an “occupied” LUMO level (L1) that is shifted downward and an unoccupied LUMO level (L2) that is shifted upward (Figure 1d, right).^16^ This downshift of the LUMO level upon charge transfer can substantially stabilize C_60_ adsorbates on graphene.^17^

### 2.2 Growth of C_60_ Thin Films on Graphene under Charge Transfer

With the in situ electrical gating of graphene (“Experimental and Methods” in the Supporting Information), we observed changes in i) the molecular interactions and assembly of C_60_ ad‐molecules and ii) the growth behavior of C_60_ crystals as the *E*_F_ of graphene gradually approached the *E*_F,c_.

First, C_60_ ad‐molecules may interact with each other on the graphene surface, depending on the relative position of the *E*_F_ of graphene and the *E*_F,c_. These distinctions can be well detected by Raman spectroscopy (Figure S5, Supporting Information). In both Raman spectra, the feature peaks of C_60_, i.e., the *A*_g_(1) mode at ≈500 cm^−1^ and the *A*_g_(2) mode at ≈1470 cm^−1^, were clearly observed.^18^ The position of the *A*_g_(2) peak indicates the number of intermolecular bonds to each C_60_ molecule, where each intermolecular bond shifts the peak by −5 cm^−1^.^19^ The peak position of the *A*_g_(2) mode of C_60_ grown on graphene with *E*_F_ < *E*_F,c_ is consistent with that reported for pristine C_60_ molecules.^18^, ^19^ However, the *A*_g_(2) peak of C_60_ grown on graphene with *E*_F_ > *E*_F,c_ was red‐shifted ≈3 cm^−1^; this change indicates that chemically bonded C_60_ dimers or oligomers were formed. This selective formation at high *E*_F_ strongly suggests that control of the *E*_F_ of graphene during C_60_ growth indeed determined the charge state of the C_60_ ad‐molecules.

The charge state of C_60_ ad‐molecules determines the formation of covalent bonds between two C_60_ molecules.^20^, ^21^ When C_60_ molecules have negative charges, the activation barrier for the bonding decreases. Therefore, graphene with *E*_F_ > *E*_F,c_ induced negative charges in C_60_ ad‐molecules, resulting in the formation of intermolecular bonds between C_60_ ad‐molecules. By contrast, on graphene with *E*_F_ < *E*_F,c_, C_60_ molecules were charge‐neutral and thus did not form covalently bonded C_60_ dimers.

**Figure 2:**
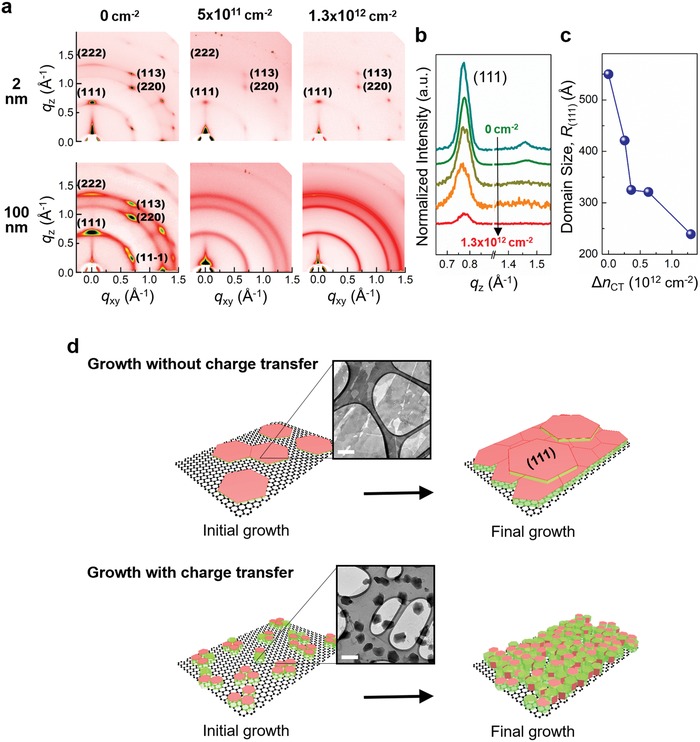
Crystal structure of C_60_ films grown on graphene. a) 2D GIXD patterns of 2.5 ML (2 nm) and thick (100 nm) C_60_ films grown on graphene when Δ*n*_CT_ = 0 cm^−2^ (left), Δ*n*_CT_ = 5 × 10^11^ cm^−2^ (middle), and Δ*n*_CT_ = 1.3 × 10^12^ cm^−2^ (right) during C_60_ deposition. b) Cross‐sectional profiles of the 2D GIXD image along the *q_z_* for various Δ*n*_CT_. c) The mean size of the crystalline (111) domains *R*_(111)_ versus Δ*n*_CT_. d) Schematic illustrations of C_60_ crystal growth on graphene without (upper) and with (lower) the charge transfer between them. Insets: Low‐magnification HR‐TEM images of corresponding graphene–C_60_ samples on TEM grids. Scale bar in insets: 200 nm.

Δ*n*_CT_ affected molecular arrangement in C_60_ crystals, and consequently, how those crystals assembled into thin films. We used grazing incidence X‐ray diffraction (GIXD) to characterize C_60_ thin films with different thicknesses grown on graphene, where Δ*n*_CT_ was controlled. Under ambient conditions, the most stable structure of C_60_ crystals is face‐centered cubic (fcc);^22^ the diffraction patterns of the fcc C_60_ were observed in our system of C_60_ thin films grown on graphene (**Figure** **2**a).

At the early growth stage (nominal thickness of 2.5 ML), irrespective of the occurrence of charge transfer, the set of reflections of (111) family and the reflections of plane (113) and plane (220) appeared; these reflections are located along the out‐of‐plane direction (*q_z_*) and at 30° and 35° tilt from *q_z_*, respectively. These results indicate that C_60_ has an epitaxial relationship with graphene, with the (111) plane of C_60_ crystals parallel to the graphene substrate;^23^ this epitaxy was independent of Δ*n*_CT_. However, differences were observed in the crystal domain sizes of C_60_ thin films grown on graphene at different Δ*n*_CT_ (Figure 2c). We quantified the average crystal domain size of C_60_ thin films by using the Scherrer equation to estimate the domain sizes of crystal plane (111) (*R*_(111)_). When Δ*n*_CT_ = 0 during C_60_ growth, C_60_ thin films had *R*_(111)_ ≈ 60 nm, which is almost three times larger than in the film grown under very high Δ*n*_CT_.

At the final growth stage, the GIXD patterns of C_60_ films grown with and without charge transfer both showed clear ring patterns, which reveal the presence of randomly oriented C_60_ crystals. However, the thick C_60_ films' ordering degree was still strongly dependent on Δ*n*_CT_. On the graphene surface where Δ*n*_CT_ = 0, the reflections were still sharp with a high signal‐to‐noise ratio, i.e., most of the C_60_ crystals were oriented. As Δ*n*_CT_ increased, these reflections weakened and eventually became undetectable; this change suggests that a large fraction of newly nucleated C_60_ crystals were randomly oriented on the pre‐existing C_60_ thin film. The growth behavior of C_60_ crystals on graphene, as indicated by GIXD experiments, is summarized as follows (Figure 2d). A highly crystalline film of fcc C_60_ was epitaxially formed on graphene via a layer‐by‐layer growth mode at negligible Δ*n*_CT_ during C_60_ growth. When Δ*n*_CT_ > 0, despite the epitaxial relationship between graphene and C_60_ at the early growth stage, randomly oriented nucleation occurred during vertical growth. These inferences are confirmed by low‐magnification high‐resolution transmission electron microscopy (HR‐TEM) images (insets of Figure 2d). At Δ*n*_CT_ = 0, large‐area C_60_ layers were observed; by contrast, at very high Δ*n*_CT_, small C_60_ clusters formed. Although the GIXD results provided a hint about the crystal structure of the C_60_ films grown on graphene over a macro area, they could not directly reveal the arrangement among C_60_ molecules and the carbon atoms in graphene.

**Figure 3:**
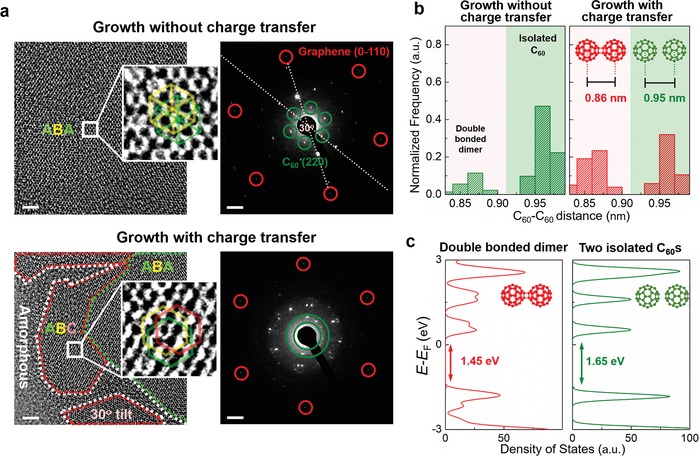
Epitaxial molecular arrangement of C_60_ on graphene. a) Typical HR‐TEM images and SAED patterns of 2.5 ML C_60_ grown on graphene when Δ*n*_CT_ = 0 (top) and Δ*n*_CT_ >> 0 (bottom). Scale bars in HR‐TEM images: 3 nm; in SAED patterns: 1 nm^−1^. Insets: High‐magnification HR‐TEM images of regions with ABA (in (top)) and ABC (in (bottom)) stacking. b) Histogram plots of nearest neighbor C_60_–C_60_ molecule distances extracted from HR‐TEM images when the growth associated without (left) and with (right) charge transfer. c) DFT energetic simulations of C_60_–C_60_ double‐bonded dimer (left) and isolated C_60_ molecules (right).

Therefore, C_60_ thin films (2.5 ML) grown on graphene were imaged at high magnification using HR‐TEM. The image of C_60_ grown on graphene at Δ*n*_CT_ = 0 clearly showed an ordered hexagonal arrangement of C_60_ molecules over a few tens of nanometers, which is the fashion of the (111) plane of a highly crystalline fcc structure (**Figure** **3**a, top). Moreover, the ordering in this HR‐TEM image matches that of ABA‐stacked C_60_ layers.^24^ This stacking order was uniform over the analyzed areas; this consistent order implies that C_60_ layers were preferentially stacked on each other in an ABA manner when the thin film was grown on graphene at Δ*n*_CT_ = 0. The corresponding selected‐area electron diffraction (SAED) pattern of this C_60_ thin film also showed only a single set of hexagonal patterns, i.e., the crystalline orientation of C_60_ was uniform along the vertical direction. Notably, when Δ*n*_CT_ = 0 was maintained during C_60_ growth, the misorientation angles between the SAED patterns of C_60_ and those of graphene were concentrated at close to 0° and 30°, which correspond to energetically stable adsorption sites of C_60_ molecules along the armchair and zigzag directions of graphene, respectively (Figure S7f, Supporting Information).^23^ This result is further evidence of an epitaxial relationship between graphene and C_60_.

By contrast, when C_60_ was grown on graphene under a very high Δ*n*_CT_, HR‐TEM image and the corresponding SAED patterns (Figure 3a, bottom) typically revealed polycrystalline C_60_ thin film along the lateral direction and vertical direction. This C_60_ film showed ABA and ABC stacking mixed within small areas. In addition, small crystalline domains were tilted from the rest with a high angle (≈30°) in this film (Figure 3a, bottom left). Notably, the areas between the tilt grains mostly exhibited an amorphous structure. On top of this amorphous region, C_60_ molecules could not arrange well, so the results were i) randomly oriented nucleation of C_60_ crystals and ii) the formation of additional amorphous layers, or both. The resulting richness of tilt grain boundaries could result in the observed polycrystallinity along both the lateral and vertical directions. The dominance of (111)‐plane‐oriented C_60_ crystal domains (Figure 2a) suggests the presence of an epitaxial relationship between C_60_ and graphene at this small thickness, so grains that have high tilt angle may be formed by stitching C_60_ domains aligned along the armchair direction and those aligned along the zigzag direction of graphene.

HR‐TEM was also the best tool to investigate the chemically bonded dimers in C_60_ films (Figure S5, Supporting Information). To quantize the dimer content, we analyzed numerous intermolecular distances of two nearest‐neighbor C_60_ molecules in films grown at Δ*n*_CT_ = 0 and Δ*n*_CT_ > 0 (Figure 3b). In both cases, the distance distribution showed peaks centered near 0.86 and 0.95 nm, but the relative peak heights depended on Δ*n*_CT_. We could assign the 0.85 nm peak to double‐bonded C_60_ dimers, and the 0.95 nm peak to isolated C_60_ molecules.^25^ When Δ*n*_CT_ = 0, more than half of the intermolecular distances were close to 0.95 nm; this consistent separation implies that a large portion of the C_60_ molecules were still free and intact. However, at Δ*n*_CT_ > 0 the fraction of free C_60_ molecules was substantially reduced and the proportion of double‐bonded dimers increased. These results qualitatively show that charge transfer with graphene during C_60_ growth promoted the formation of double‐bonded C_60_ dimers.

In addition, we performed density functional theory (DFT) simulations to calculate the electronic structure of a double‐bonded C_60_ dimer and two isolated C_60_ molecules (Figure 3c; Figure S14, Supporting Information). Compared with isolated C_60_ molecules, a double‐bonded C_60_ dimer showed an ≈0.2 eV smaller bandgap, and broader LUMO and highest occupied molecular orbital (HOMO) levels.

**Figure 4:**
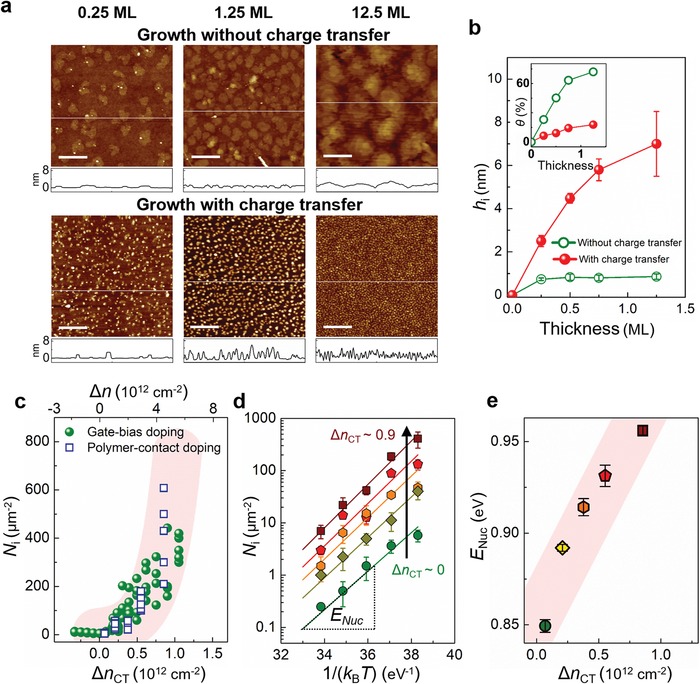
Nucleation of C_60_ islands on graphene. a) AFM images of C_60_ at different nominal thicknesses of 0.25 ML (left), 1.25 ML (middle), and 12.5 ML (right) grown on graphene, without (upper) and with (lower) the charge transfer. Scale bar: 400 nm. b) Height analysis for C_60_ islands in the AFM images. Inset: Surface coverage analysis. c) Nucleation density *N*_i_ versus Δ*n*_CT_ from gate‐bias (green circle) and polymer‐contact doping (blue square). d) *N*_i_ versus thermal parameter 1/(*k*_B_ *T*). e) Nucleation energy barrier of C_60_ (*E*_Nuc_) versus Δ*n*_CT_ calculated from (d). Shaded areas are to guide the eye.

The effects of charge transfer on C_60_ growth behaviors are further demonstrated by morphological analysis using atomic force microscopy (AFM) (**Figure** **4**a), which enabled statistical analysis of average height *h*_i_ of C_60_ islands and surface coverage θ of the thin films during the early growth stage (Figure 4b). On the surface of graphene templates on which charge transfer was suppressed, i.e., *E*_F_ < *E*_F,c_, the initial large‐area C_60_ islands expanded laterally, to yield a constant monolayer thickness (0.8 nm) and a large increase of surface coverage. As electron transfer from the graphene to C_60_ ad‐molecules increased, the number of nuclei quickly increased and each of them merely grew in height; the result was an array of grains of different heights. At Δ*n*_CT_ = 0, as the growth continued, continuous C_60_ film was formed by coalescence of large‐area C_60_ grains; by contrast, at Δ*n*_CT_ > 0, C_60_ film was formed by full coverage of small C_60_ islands with poor inter‐grain connection. At the later growth stage (12.5 ML), the C_60_ thin film grown at Δ*n*_CT_ = 0 revealed clear terrace structure, which is evidence of lateral growth mode, whereas the film grown at Δ*n*_CT_ > 0 simply showed an array of tiny crystallites.

The charge transfer in the graphene–C_60_ system as well as its effects on the crystal structure and morphology of C_60_ (Figures 2, 3, 4) were elucidated using electrically gated graphene templates. The use of polymer–substrate‐doped graphene revealed similar results (Figures S1, S6, and S9, Supporting Information). This comparison emphasizes that other factors (e.g., localized traps, the wetting transparency, or contamination on the graphene surface) that might obscure the collected results might have been effectively eliminated.^7^ Moreover, this polymer–substrate doping method could provide a general understanding of the observed phenomena.

To quantify the dependence of C_60_ growth on the charge transfer from the graphene template to C_60_ ad‐molecules, numerous C_60_ thin films with a nominal thickness of 0.25 ML were grown on graphene templates whose *E*_F_ was finely controlled by either gating or polymer–substrate doping. The plot of the nucleation density (*N*_i_) of these films against Δ*n*_CT_ at room temperature revealed correlations between the nucleation of C_60_ and charge transfer from graphene to C_60_ (Figure 4c; Figure S10, Supporting Information). Clearly, *N*_i_ increased as Δ*n*_CT_ increased. We also directly measured the activation energy for C_60_ nucleation (*E*_Nuc_) as a function of Δ*n*_CT_, as *N*_i_ = *C* exp (*E*_Nuc_/(*k*_B_ *T*)) where *C* is a pre‐exponential factor, *k*_B_ is the Boltzmann constant, and *T* is the substrate temperature.^26^ To this end, *N*_i_ values as a function of the substrate temperature *T* were collected at various fixed Δ*n*_CT_; the slopes of plots of ln(*N*_i_) versus 1/(*k*_B_ *T*) at a certain Δ*n*_CT_ gave the values of *E*_Nuc_ at the Δ*n*_CT_ (Figure 4d). As a result, we confirmed that *E*_Nuc_ increased as Δ*n*_CT_ increased (Figure 4e).

### 2.3 Atomistic Mechanism of C_60_ Thin Film Growth on Graphene under Charge Transfer

**Figure 5:**
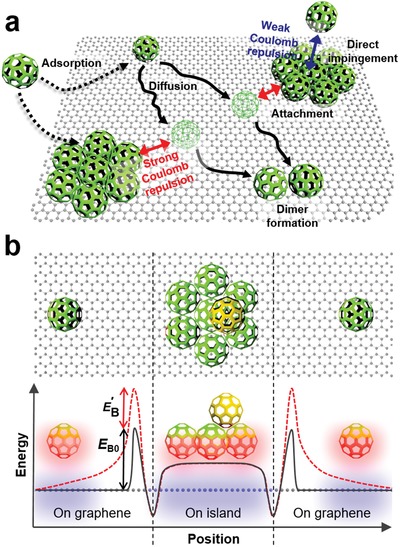
Mechanism of C_60_ growth on graphene. a) Nucleation process of C_60_ crystals on graphene surface that involves adsorption, diffusion, dimer formation, attachment, and direct impingement. b) Energy profiles of a C_60_ ad‐molecule versus position near and on a C_60_ island under the absence (solid line) and presence (dashed line) of the charge transfer between the ad‐molecule and graphene.

The nucleation of C_60_ on graphene involves several atomistic processes (**Figure** **5**a). After adsorbing to graphene, an C_60_ ad‐molecule diffuses on the surface until the molecule forms a dimer with another ad‐molecule or attaches to a pre‐existing island (growth).^27^, ^28^ In general, *E*_Nuc_ is related to the activation energies of all of these atomistic processes. However, the energy barrier for C_60_ diffusion is negligible on graphitic surfaces,^24^, ^29^ so nucleation and growth of C_60_ on graphene are predominantly limited by the rate of attachment of ad‐molecules to pre‐existing islands.

For such attachment‐limited nucleation with negligible barriers to diffusion and dimerization, *E*_Nuc_ = [2*E*_i_ + 2(*i* + 1)*E*_B_]/(*i* + 3),^27^ where *i* is critical cluster size, *E*_i_ is cluster energy, and *E*_B_ is activation energy for the attachment.^27^ This equation implies that a nucleation density increases as *E*_B_ increases. This relation is explained as follows. The presence of high *E*_B_ hinders the attachment of deposited ad‐molecules to an island, so the concentration of ad‐molecules increases on the graphene surface. Thus, the probability of ad‐molecules colliding rapidly increases, and this change favors new nucleation rather than the growth of pre‐existing islands.

Therefore, the increases in *N*_i_ and *E*_Nuc_ with increasing Δ*n*_CT_ are attributable to the increase in *E*_B_ with increasing Δ*n*_CT_ as *E*_B_ (Δ*n*_CT_) = *E*_B0_  + *E*′_B_(Δ*n*_CT_) where *E*_B0_ is the charge‐transfer‐independent attachment barrier and *E*′_B_ is the charge‐transfer‐dependent attachment barrier. When electrons in graphene are transferred to the ad‐molecules and the islands, the ad‐molecules and islands are negatively charged and the underlying graphene becomes positively charged (Figure 5b; Figure S11, Supporting Information). Consequently, repulsive Coulomb interaction occurs between the dipole from the ad‐molecule–graphene and that from the island–graphene. This long‐range repulsive interaction would introduce an additional attachment barrier *E*′_B_. Assuming the long‐range repulsive interaction is simple electrostatic repulsive interaction, *E*′_B_ can be estimated as

(4) $$E\prime_{\text{B}}^{}=Z_{\text{avg}}\;e^{2}d\Delta n_{\text{CT}}\text{/}2\varepsilon_{0}$$

where *Z*_avg_ is the average charge state of C_60_ ad‐molecules (Equation (4) is derived in the Supporting Information). This model successfully predicts the increase in *E*_B_ with increasing Δ*n*_CT_. In this argument, we assumed that repulsive Coulomb interaction between an ad‐molecule and an island (and not that between two ad‐molecules on graphene) dominantly affects the nucleation kinetics. This assumption can be justified because the probability of collision between two C_60_ molecules which both simultaneously have negative charges would be very small. On the contrary, a C_60_ island contains many C_60_ molecules, so a C_60_ island is likely negatively charged.

The transition from a 2D to a 3D growth mode (Figures 2, 3, 4) under the charge transfer between graphene and C_60_ can be simply explained by invoking the repulsive Coulomb interaction between an ad‐molecule and an existing island. With increasing Δ*n*_CT_, *E*_B_ increases because of the repulsive interaction; this change inhibits the lateral growth of negatively charged islands by negatively charged ad‐molecules diffusing on the graphene surface. However, irrespective of the *E*_F_ of the graphene template, the ad‐molecules from the vapor phase can land directly on the top of the existing island because they are charge‐neutral and thus not prone to the repulsive Coulomb interaction. However, after they are deposited on the top of the islands, their dynamics are again influenced by the *E*_F_ of graphene. When Δ*n*_CT_ = 0, they can move relatively freely down to the graphene surface because the Ehrlich–Schwoebel barrier is much lower than the diffusion barrier on top of the C_60_ layer.^28^ When electrons are transferred from graphene to C_60_, the *E*_B_ increases and thus acts as an energy wall surrounding the edge of islands. For an ad‐molecules on the top of the island to move downward and escape from the island, they must overcome an activation energy greater than *E*_B_. Consequently, ad‐molecules become concentrated on the top of the island, so the island rapidly grows in the vertical direction. The rapid vertical growth in turn leads to the formation of randomly oriented crystals.

### 2.4 Charge Transport in C_60_ Thin Films and Graphene–C_60_ Junctions

**Figure 6:**
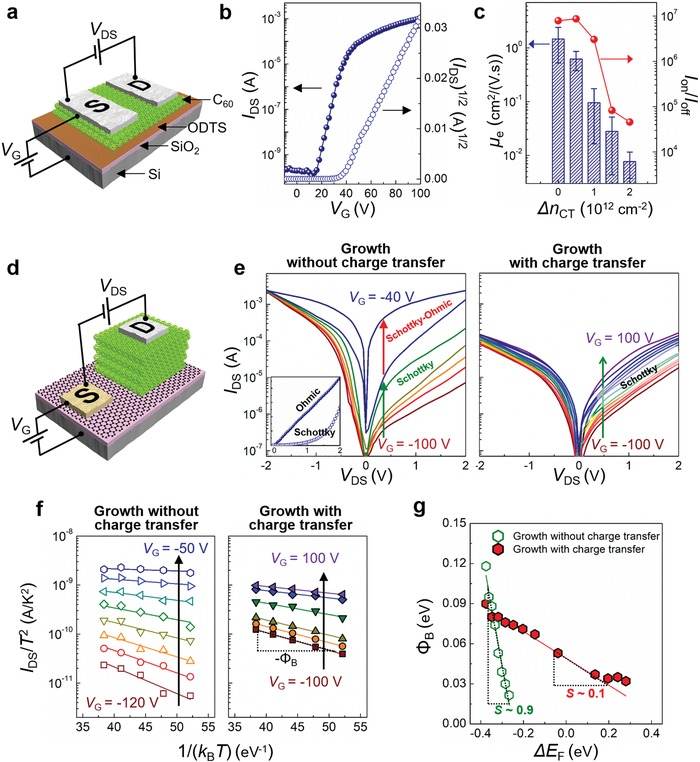
C_60_ field‐effect transistors and graphene–C_60_ barristors. a) Schematic illustration of planar C_60_‐FET. b) Transfer characteristic of C_60_‐FET with C_60_ film grown at Δ*n*_CT_ = 0. c) Average *I*_on_/*I*_off_ and electron mobilities μ_e_ of C_60_‐FETs versus Δ*n*_CT_ during C_60_ growth. d) Schematic illustration of graphene–C_60_ barristor. e) *I*_DS_ versus *V*_DS_ of graphene–C_60_ barristors at various fixed *V*_G_ (from −100 to −40 V, step 10 V) for Δ*n*_CT_ = 0 (left) and at *V*_G_ (from −100 to 100 V, step 10 V) for Δ*n*_CT_ > 0 (right). Inset: *I*_DS_ versus *V*_DS_ at linear scale of graphene–C_60_ barristor for Δ*n*_CT_ = 0 at *V*_G_ = −40 V (filled symbols) and *V*_G_ = −30 V(open symbols). f) Temperature‐dependent saturation current of graphene–C_60_ barristors at various *V*_G_ for Δ*n*_CT_ = 0 (left, step 10 V) and Δ*n*_CT_ > 0 cases (right, step 40 V). g) The Schottky barrier height (Φ_B_) obtained from (f) versus Δ*E*_F_.

To quantify the advantage of our growth‐controlled C_60_ thin films for lateral charge transport, we grew C_60_ thin films on graphene at controlled charge‐transfer conditions, then transferred the C_60_ films to octadecyltrichlorosilane (ODTS)‐treated SiO_2_/Si substrates and then fabricated planar C_60_ transistors (C_60_‐FETs). The final device included an ≈50 nm thick C_60_ channel without the underlying graphene (**Figure** **6**a). We measured the transfer characteristics of C_60_‐FETs in the saturation regime with C_60_ thin‐film channels grown at different Δ*n*_CT_, then estimated the associated electron field‐effect mobility (μ_e_) and measured the on/off ratio (*I*_on_/*I*_off_). For a C_60_ thin film grown at Δ*n*_CT_ = 0, the *I*_on_/*I*_off_ of the FET device was ≈10^7^ and the average μ_e_ was ≈1.5 cm^2^ V^−1^ s^−1^. The maximum mobility of the device was ≈2.5 cm^2^ V^−1^ s^−1^, which is similar to the state‐of‐the art mobility of C_60_ transistors fabricated by the vapor deposition method (Figure 6b).^5^, ^30^ With increasing Δ*n*_CT_, the *I*_on_/*I*_off_ and μ_e_ of the device substantially decreased, and eventually reached the same level of devices fabricated with polycrystalline and small‐grain C_60_ (Figure 6c).^31^ The decay occurs because the high Δ*n*_CT_ causes low crystallinity, low uniformity and limited grain size, and these traits suppress the lateral μ_e_ of C_60_ thin films.

Our method of growing C_60_ thin films on graphene provides a direct way to produce controlled graphene–C_60_ van der Waals heterostructures. In addition to its use as a growth template, graphene can function as an active layer or electrode for various flexible optoelectronic devices because of its excellent electrical conductivity and flexibility. Recently, heterostructures composed of graphene and OSCs have shown promise for use in organic photovoltaics, organic light‐emitting diodes, organic photodetectors, and vertical FETs.^4^, ^5^ The electrical characteristics of such devices depend on the charge‐injection efficiency at the graphene–OSC interface.

Such charge‐injection efficiency at the graphene–C_60_ van der Waals heterointerface formed with our method was demonstrated by fabricating two types of graphene–C_60_ barristors. They had the same device structure, but one had C_60_ film grown at Δ*n*_CT_ = 0, and one had C_60_ film grown at Δ*n*_CT_ = 1 × 10^12^ cm^−2^ (Figure 6d), so the C_60_ layers had enormously different morphological and crystalline features. Both devices showed typical n‐type barristor behavior (Figure 6e).^1^ The closeness between the LUMO level of C_60_ and Fermi level of aluminum yields Ohmic contact between the C_60_ and the top aluminum electrode,^32^ so rectifications arose from the Schottky barrier (Φ_B_) between the C_60_ layer and the bottom graphene. Increasing the *V*_G_ barely affected the current in the forward regime (*V*_DS_ < 0) but boosted the current in the reverse regime (*V*_DS_ > 0). Consistent with the band diagram (Figure 1), the increase of *V*_G_ raised the Fermi level of graphene closer to the LUMO level of C_60_, reducing the Φ_B_ accordingly, until alignment was achieved between them (Φ_B_ ≈ 0, Ohmic contact).

Although both barristors showed rectification behavior, great distinction was observed in the current levels between the two devices. The device that used the C_60_ layer that had been grown at Δ*n*_CT_ = 0 showed substantial modulation of the reversed current by the gate voltage; and the on‐state current *I*_on_ was higher in this device than in the device that used the C_60_ layer that had been grown at Δ*n*_CT_ > 0, whereas their off‐state currents *I*_off_ were similar. As a result, this device fabricated with a highly crystalline C_60_ film (i.e., grown at Δ*n*_CT_ = 0) achieved an *I*_on_/*I*_off_ ratio of ≈10^3^ at *V*_DS_ = 2 V, which is nearly two orders of magnitude greater than the *I*_on_/*I*_off_ ratio of the other device at the same *V*_DS_ (Figure 6e; Figure S12, Supporting Information).

The most important difference between the two types of barristors was the occurrence of a Schottky‐to‐Ohmic transition, which was only observed in the device that used the C_60_ layer that had been grown at Δ*n*_CT_ = 0 (Figure 6e, left). This transition occurred at *V*_G_ = −40 V, which is consistent with the critical voltage (*V*_G_ at *n*_c_) required to induce charge transfer between graphene and C_60_ (Figure 1). By contrast, Schottky‐to‐Ohmic transition was not observed within the wider examining *V*_G_ range for the device with the C_60_ layer grown at Δ*n*_CT_ > 0 (Figure 6e, right); this absence implies that modulation of the *E*_F_ of graphene by electrical gating was limited at the graphene–C_60_ interface.

Fermi‐level pinning can occur when there are interfacial states in the HOMO–LUMO gap of C_60_ layers near graphene.^33^ To quantitatively analyze the Fermi‐level pinning, we used the diode equation in the reverse bias saturation regime, $I_{\text{DS}}\propto T^{2}\exp\left(-\frac{e\Phi_{\text{B}}}{k_{\text{B}}T}\right)$.^1^ The value of Φ_B_ at each *V*_G_ was then estimated from the plot of ln(*I*_DS_/*T* ^2^) versus 1/(*k*_B_ *T*) (Figure 6f). Φ_B_ increased with increasing *E*_F_ at different rates in the two device types (Figure 6g). For the barristor with the C_60_ layer grown at Δ*n*_CT_ = 0, the slope *S* = dΦ_B_/d*E*_F_ was ≈0.9, which indicates that the graphene–C_60_ junction in this device approached the Schottky–Mott limit.^14^, ^34^ This result demonstrates an atomically clean interface between graphene and the C_60_ thin film, which has not been previously achieved.^23^, ^35^ To achieve this clean heterointerface for the effective tuning of the Schottky barrier, C_60_ must be deposited directly on a thermally cleaned graphene surface,^36^ and the electronic state of graphene must be optimized to effectively limit the charge transfer during growth to enable growth of high‐crystallinity C_60_ film at the interface with graphene. The latter effect of charge transfer during the growth of OSCs has been neglected previously.

For the other device, *S* was only 0.1, which is indicative of strong Fermi‐level pinning effect at the graphene–C_60_ interface. The C_60_ thin film grown on graphene with charge transfer had small and poorly connected C_60_ grains near the graphene surface (Figures 2, 3, 4), so the interface had i) a high density of C_60_ grain boundaries, ii) large amorphous areas, and iii) other crystalline defects that would introduce numerous interfacial trap states (Figure S13c, Supporting Information). The Fermi level of graphene was pinned at those states. In addition, because the DFT results reveal a smaller bandgap of a C_60_ dimer compared with two isolated C_60_ molecules (Figure 3c), the presence of C_60_ dimers would introduce shallow charge traps, which can further contribute to the observed Fermi‐level pinning at the graphene–C_60_ interface.

To directly confirm the interfacial states between the graphene and the C_60_, photocurrents of G‐FETs fabricated with deposited C_60_ thin films (20 nm) were measured under light illumination at 0.62 eV (Figure S13a, Supporting Information). For comparison, C_60_ thin films were grown on top of graphene channels under Δ*n*_CT_ = 0 and Δ*n*_CT_ > 0. At a high positive gate bias (*V*_G_ = 80 V), only the G‐FET with the C_60_ thin film grown at Δ*n*_CT_ > 0 showed additional photocurrent as the device was illuminated (Figure S13b, Supporting Information). The excitation energy is much smaller than the bandgap of the C_60_ thin film and smaller than 2|*E*_F_| of graphene at *V*_G_ = 80 V, so the interband transitions are forbidden in both the C_60_ thin film and the graphene.^37^ Therefore, the photocurrent in the G‐FET with a C_60_ thin film was merely a result of detrapped electrons from the interfacial states, which were abundant in the layer grown at high Δ*n*_CT_. In fact, we observed positive photocurrent from the G‐FET with the C_60_ film grown at Δ*n*_CT_ > 0, but observed no photoresponse from the device with C_60_ film grown at Δ*n*_CT_ = 0.

## 3 Conclusion

We observed that charge transfer within the graphene–C_60_ system during the growth of C_60_ crystals on a graphene template governed such growth and, thus governed the thin film's corresponding crystal structure and morphology. These charge‐transfer phenomena altered the electronic states of the graphene–C_60_ system, forming negatively charged C_60_ nuclei and ad‐molecules. Under these conditions, the growth of C_60_ on graphene was favored in the vertical dimension because of the high attachment barrier energy, resulting thin films with small and randomly oriented crystallites. With this understanding, we proposed that the optimized graphene template for layer‐by‐layer growth of C_60_ with large and uniformly oriented crystals is the graphene in which the charge transfer from graphene to C_60_ is suppressed during the C_60_ growth. Barristors fabricated with this graphene–C_60_ van der Waals heterostructure showed efficient tunability of the charge injection barrier, approaching the Schottky–Mott limit. In addition, the lateral electron mobility μ_e_ in a planar C_60_‐FET was also boosted to a maximum μ_e_ = 2.5 cm^2^ V^−1^ s^−1^.

## Conflict of Interest

The authors declare no conflict of interest.

## Supporting information

Supporting Information
