# Supplementary material for: Charge‐Transfer‐Controlled Growth of Organic Semiconductor Crystals on Graphene
Source: Adv Sci (Weinh). 2020 Feb 14;7(6):1902315. doi: 10.1002/advs.201902315 (PMC7080519; doi:10.1002/advs.201902315)
Supplement: Supplementary file 1 — Supporting Information [file ADVS-7-1902315-s001.pdf]

Copyright WILEY-VCH Verlag GmbH & Co. KGaA, 69469 Weinheim, Germany, 2020.

ADVANCED SCIENCE
Open Access

Supporting Information

for *Adv. Sci.,* DOI: 10.1002/advs.201902315

Charge-Transfer-Controlled Growth of Organic Semiconductor Crystals on Graphene

*Nguyen Ngan Nguyen, Hyo Chan Lee, Min Seok Yoo, Eunho Lee, Hansol Lee, Seon Baek Lee, and Kilwon Cho**

Copyright WILEY-VCH Verlag GmbH & Co. KGaA, 69469 Weinheim, Germany, 201p.

Supporting Information

Charge-Transfer-Controlled Growth of Organic Semiconductor Crystals on Graphene

*Nguyen Ngan Nguyen, Hyo Chan Lee, Min Seok Yoo, Eunho Lee, Hansol Lee, Seon Baek Lee, and Kilwon Cho**

Experimental and methods

Graphene growth and transfer processes. We controlled the Fermi level E_F of graphene by using two methods of doping: electrical gate doping (Figure 1a in the main text) and polymer-contact doping (Figure S1), which have been described previously.[1] Graphene was grown on a copper foil by chemical vapor deposition (CVD), as described elsewhere.[2] The final product was either graphene/300-nm SiO_2/*p*-doped Si for electrical-gate-doped graphene templates, or graphene/polymer/300-nm SiO_2/*p*-doped Si for polymer-contact doped graphene templates. The polymers used were (1) poly(4-vinylphenol) (P4VP) and poly(4-vinylpyridine) (P4VPh) to induce *n*-doping in graphene; (2) poly(methyl methacrylate) (PMMA) and poly(vinyl chloride) (PVC) to induce *p*-doping in graphene; and (3) polystyrene (PS) to keep graphene in pristine electronic state.

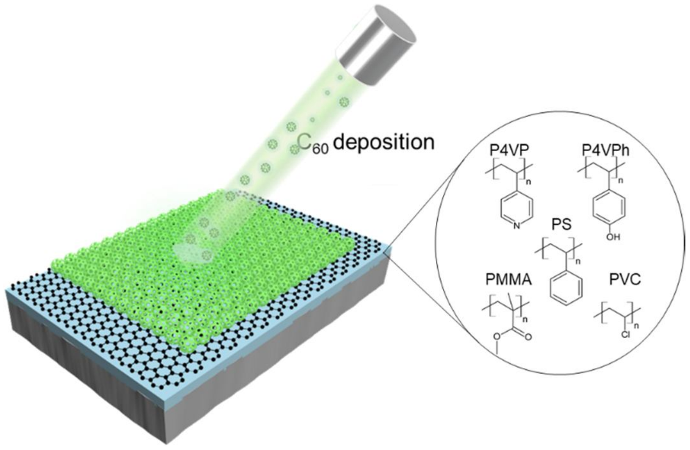

Figure S1. Schematic illustration of polymer-doped graphene for C_{60} growth template.

Graphene characterizations. The doping effects of polymers on graphene have been described previously.[1] Briefly, the charge-carrier density n_g in polymer-contact doped graphene was 1.7 × 10^{12} cm^{-2} with P4VP, 7.2 × 10^{11} cm^{-2} with P4VPh, ~0 with PS, -1 × 10^{12} cm^{-2} with PMMA and -2.7 × 10^{12} cm^{-2} with PVC.

C_{60} deposition. Graphene/SiO_2/Si was annealed at 350 °C in H_2 atmosphere for 1 h with slow cooling to remove the polymeric residues before further use. An organic molecular beam deposition (OMBD) system was used to deposit C_{60} (Aldrich Chemicals, 99.99% purity) in ultra-high vacuum (UHV, 10^{-8} Torr). The deposition rate, film thickness, and substrate temperature mentioned in the main text were the values recorded by the monitor. Throughout the experiment, the substrate was kept at 303 K, and C_{60} was deposited onto it at a rate of 5 × 10^{-2} monolayer per second (ML/s) unless stated otherwise.

To achieve the charge transfer control during the preparation process, we had to apply gate voltage to graphene during C_{60} deposition. To do that, we deposited an Au electrode pad on a graphene that had been transferred onto a 300-nm-thick SiO_2/ *p*-doped Si wafer. Heavily-doped silicon was used as a bottom gate electrode. Then a electrical potential difference between the Au electrode and Si was generated using power sources. The voltage was controlled by connecting power sources in series and was checked before each C_{60} deposition. To prevent possible charge accumulation in the sample, we used a Cu wire to connect the Au electrode pad to the conducting part of OMBD system. For safety reason, the applied voltage was kept < 100 V.

Discussion

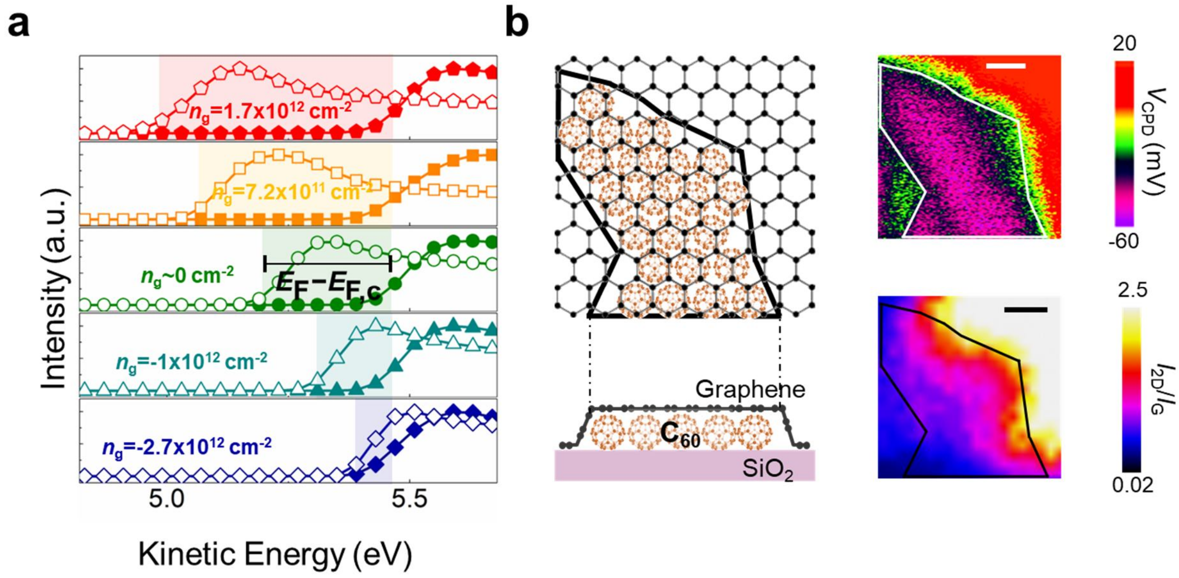

Figure S2. Graphene properties after deposition of 1 ML C_{60}. a, UPS measurement in the SEC region of graphene with different charge carrier densities before (open) and after (filled) C_{60} deposition. **b,** Schematic illustration of graphene-C_{60} system for the following experiments: KPFM (top) and Raman mapping (bottom).

Qualitative estimation of charge transfer between graphene and C_{60}. Charge transfer between graphene and C_{60} was qualitatively observed by tracing the changes in graphene properties by using ultraviolet photoelectron spectroscopy (UPS, 4D-beamline, Pohang Accelerator Laboratory (PAL), Korea), Raman spectrometry (Alpha300R, WITec, λ = 532 nm), and Kelvin probe force microscopy (KPFM) (Figure S2).

UPS measurements in the secondary electron emission region (SEC) were performed on graphene/polymer samples before and after deposition of 1 ML C_{60} (Figure S2a). After C_{60} deposition, E_F of graphene/C_{60} was pinned at the lowest unoccupied molecular orbitals (LUMO) level of C_{60}. Such pinning can be detected by the n_g-independent SEC-spectrum of graphene/C_{60}. The *p*-doping effect of C_{60} on graphene was clearly demonstrated by the right-

shifts of the UPS spectra. The degree of shift increased as n_g increased; this trend means that as the E_F of graphene approached the LUMO of C_{60}, the (E_F - $E_{F,c}$) increased, so the doping effect increased.

For Raman mapping and KPFM measurement, 1 ML C_{60} was deposited through a shadow mask onto freshly-grown graphene/copper which was *n*-doped. The C_{60}/graphene/copper was transferred using the conventional transfer process, then flipped over onto a substrate so that the upper surface was clean graphene (Figure S2b). This structure ensures that the collected results were from graphene only, not from the C_{60} layer. A KPFM image clearly showed the difference of V_{CPD} between the clean graphene area and the graphene area with the underlying C_{60} layer; this change implies that graphene's work function was shifted as coupled with C_{60}.

For Raman mapping, we optimized the scan conditions so that the spectra of C_{60} and graphene did not mix. The scan was performed over the area that we had examined using KPFM. The intensity ratio (I_{2D}/I_G) of the 2D peak (I_{2D}) and G peak (I_G) revealed the doping intensity of graphene. The clean graphene area showed a very high I_{2D}/I_G ~ 2.5, which indicates that the graphene was undoped. In the area with the underlying C_{60}, I_{2D}/I_G was significantly reduced; this result confirms that C_{60} had doped the graphene.

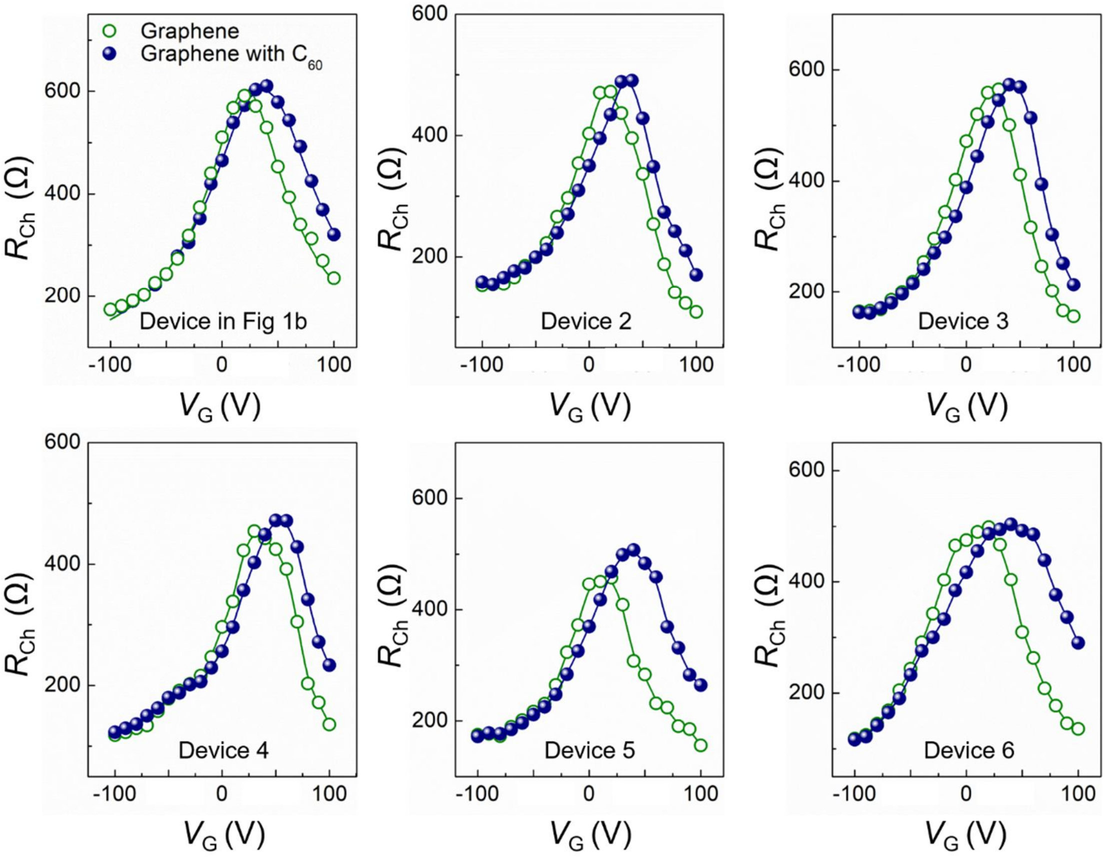

Figure S3. Transfer characteristic of various G-FETs that used graphene from different batches before (green open circle) and after C_{60} deposition (blue closed circle).

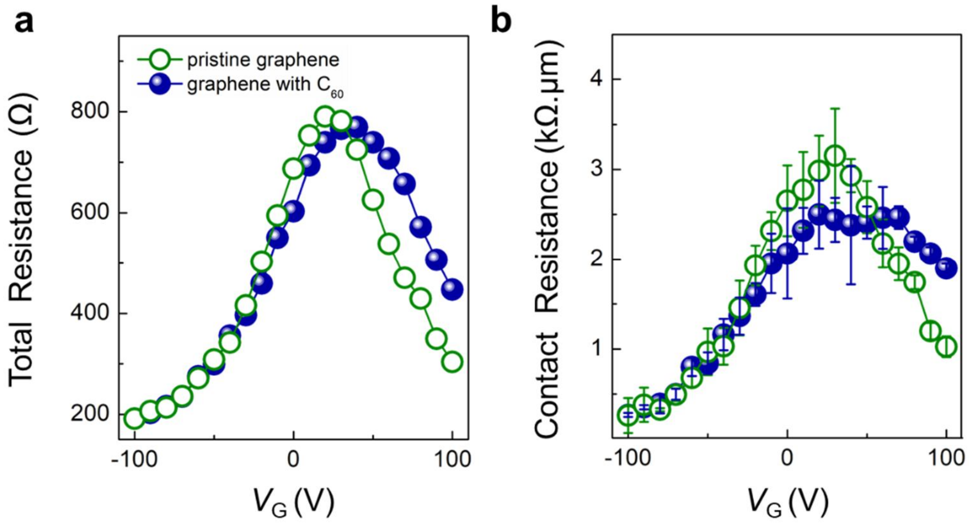

Figure S4. **TLM measurements of graphene channels before and after deposition of C_{60}**. **a,** Total resistance of graphene vs. applied gate voltage V_G. **b,** Contact resistance of graphene vs. applied gate voltage V_G.

Quantitative estimation of charge transfer between graphene and C_{60}. Charge transfer between C_{60} and graphene was quantified by measureing changes in resistance. Graphene's resistance without contact effects was measured using transfer-length method (TLM); and the changes in graphene channel resistance before and after 3 s of C_{60} deposition at a deposition rate 5×10^{-2} ML/s were used to calculate the charge transfer number (Figure 1 in the main text and Figure S3, S4).

To minimize contact resistance R_C, annealed graphene/SiO_2/p-Si was exposed to UV–O_3 for 5 s right before depositing 100 nm of gold (Au) with shadow masks. Au was evaporated at a low rate of 0.2 Å/s. The shadow mask consisted of five channel lengths: 30, 80, 130, 180, and 280 µm. A second shadow mask was used with reactive-ion etching to achieve graphene stripes with 30 µm width. The measurements were conducted under high vacuum (10^{-6} Torr).

V_G–dependent R_C of graphene transistors is inversely proportional to either the lowest number of conduction modes M_m in the graphene under the metal electrodes, or to the lowest number of conduction modes M_{ch} in the graphene channel; i.e., $M_{min} = \min\{M_m, M_{Ch}\}$. Here, M is obtained as $M = W|\Delta E_F|/\pi\hbar v_F$ where W is the channel width, ΔE_F is the change in graphene's Fermi level, □ is the reduced Planck's constant, v_F is the Fermi velocity of graphene. Therefore, $R_C(V_G)$ is proportional to $min\{\Delta E_{F,m}(V_G), \Delta E_{F,Ch}(V_G)\}^{-1}$.[3]

Before C_{60} deposition, the peak of R_C was located at the Dirac voltage of a graphene FET; this result indicates that $\Delta E_{F,m} > \Delta E_{F,Ch}$ in the range of measured V_G. This relationship probably occurred because E_F of graphene under a metal electrode was pinned by the metal. This effect simplifies the interpretation of V_G–dependent R_C after C_{60} deposition. Graphene regions under the metal electrodes were not affected by C_{60} deposition, so the change in R_C

upon C_{60} deposition was solely attributed to the change in $\Delta E_{F,Ch}$, i.e., $R_C \propto {\Delta E_{F,Ch}}^{-1}$. As a result, the R_C–V_G curves of graphene transistor before and after C_{60} deposition showed the same trend as the R_{Ch}–V_G curves of graphene transistors (Figure 1b in the main text); this similarity confirms our results.

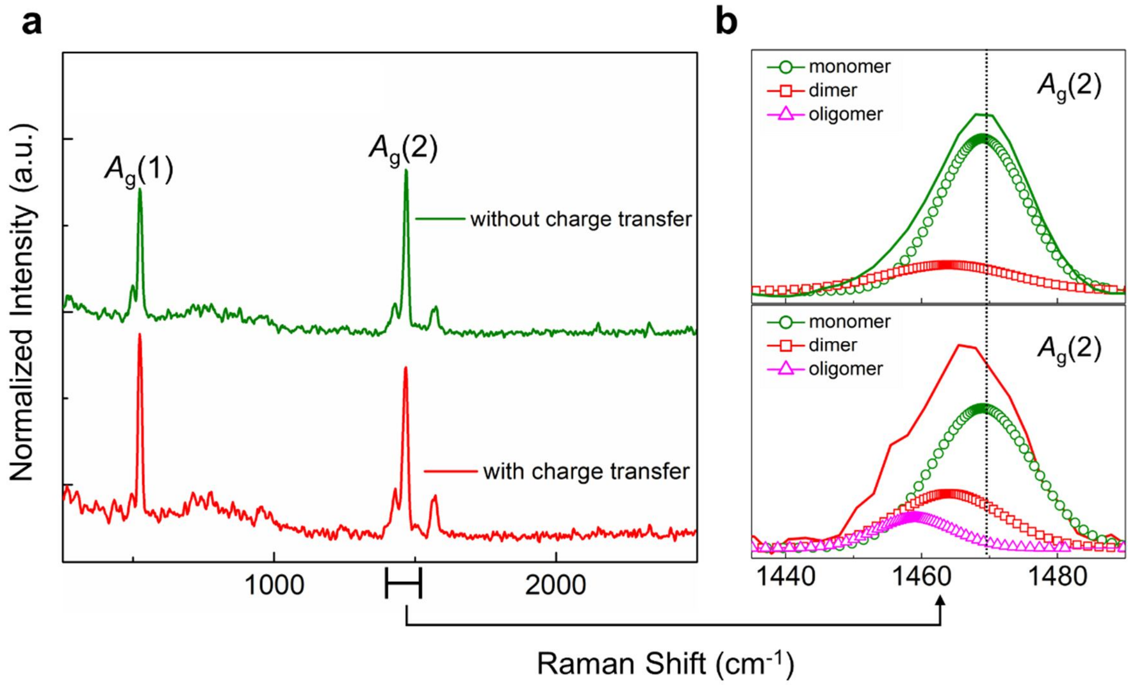

Figure S5. **Raman measurement of C_{60} thin films grown on graphene**. **a,** Full Raman spectra. **b,** Enlargement of $A_g(2)$ mode.

Raman spectra of C_{60} thin films. C_{60} thin films of 20-nm thickness were deposited onto graphene with $\Delta n_{CT} \sim 0$ which corresponds to lack of charge transfer, and onto graphene with $\Delta n_{CT} >> 0$ which corresponds to presence of charge transfer. The laser's wavelength was 532 nm and was focused onto C_{60} layer so that graphene's signal was phased out (Figure S5). The spectra were collected with low accumulation to limit damage to the sample.

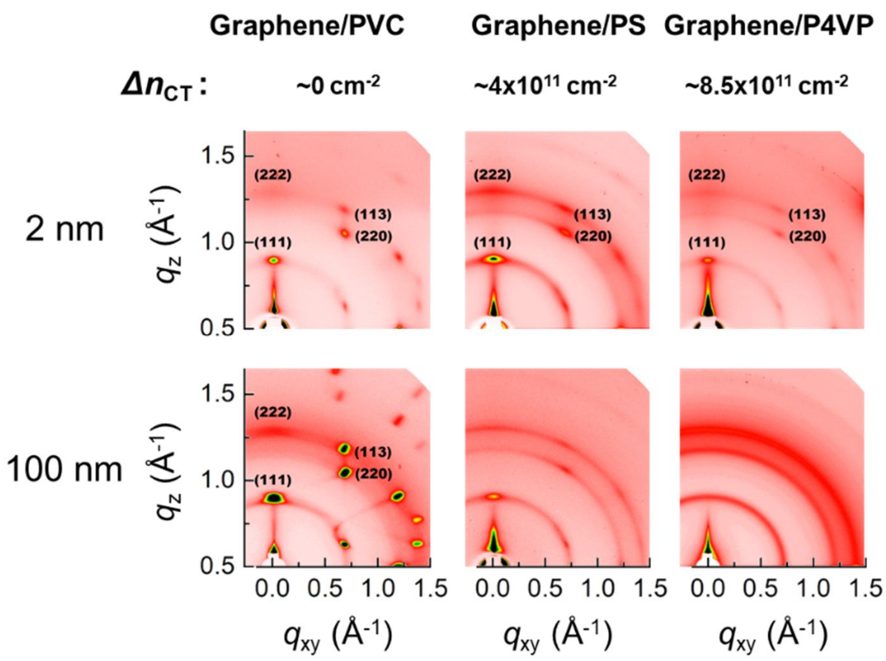

Figure S6. **Crystal structure of C_{60} thin films grown on graphene.** GIXD patterns of C_{60} thin films, 2 nm and 100 nm thick, grown on polymer-contact doped graphene.

Crystal structure of C_{60} films grown on graphene. Crystal structure of C_{60} films was characterized using grazing incident x-ray diffraction (GIXD, 3C and 9A beamlines in PAL) and transmission electron microscopy (TEM, HR-FE-TEM-2200FS with Cs correction).

For the general observation, both electrical-gate doped and polymer-contact doped graphene templates were used separately for growth of C_{60} crystals. The crystal structure of C_{60} and the trend of results on both kinds of graphene templates were similar (Figure 2 in the main text and Figure S6); this observation confirms the effects of charge transfer on the crystal structure of C_{60} films.

The coherent size of *(111)* domain was calculated using the Scherrer equation $R_c = \frac{K\lambda}{\Gamma\cos(\theta_{Bragg})}$, where K is the dimensionless Scherrer constant, λ is the incident X-ray wavelength, Γ is the full-width at half-maximum of the corresponding reflection, and θ_{Bragg} is its Bragg angle.

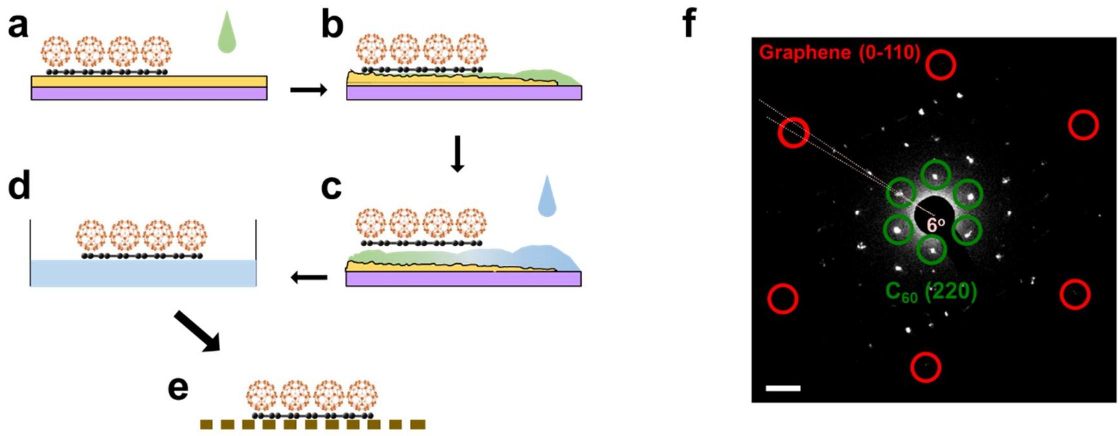

Figure S7. Sample preaparation for TEM measurement and additional SAED pattern. **a**, Dropping Au etchant on Au film and outside the area of sample; **b**, Au etchant diffuses under the sample area during etching process; **c**, dilute Au etchant with DI water; **d**, rinsing the sample with DI water; **e**, scooping up the sample with TEM grid. **f**, Additional SAED pattern

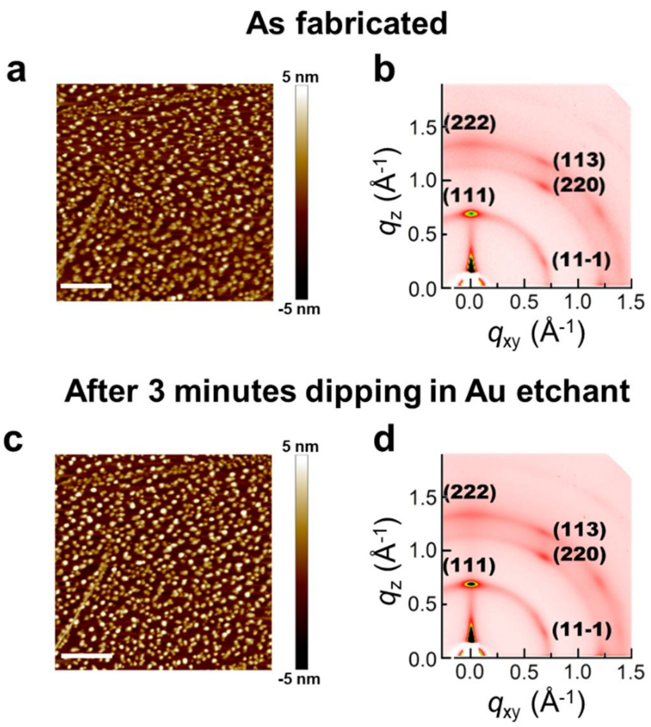

of C_{60} grown on graphene without charge transfer. Scale bar: 3 nm.

Figure S8. Insignificant effects of Au etchant on C_{60}/graphene samples. AFM image (a) and GIXD pattern (b) of 1.25-ML C_{60}/graphene before and after dipping in Au etchant for 3 min (c, d). Scale bar: 400 nm.

For TEM measurements, 2 nm of C_{60} was grown on graphene/300-nm SiO_2/Si and 2-nm C_{60}/graphene bilayer was transferred onto 50-nm Au/SiO_2/Si by PDMS stamping. To etch sacirificial Au layer, we dropped very small amount of the etchant (0.02 ml 0.01M KI/I_3) outside the C_{60}/graphene area, and let it naturally diffuse to the Au area under the C_{60}/graphene area. Once the Au layer was dissolved, DI water was immediately but tenderly dropped on the sample to rinse the etchant. Then, C_{60}/graphene was scooped using by a piece of polyethylene terephthalate (PET) and transferred to DI water for further rinsing. We scooped the floating sample on water by using a TEM grid (Lacey Carbon, copper, Ted Pella). Lastly, the sample was dried in ambient air and stored in UHV before measurement (Figure 3 in the main text and Figure S7).

To check whether the gold etchant damaged the C_{60}/graphene samples, we compared the morphology and crystal structure of 1.25-ML C_{60}/graphene/300-nm SiO_2/Si before and after dipping the sample in gold etchant solution for 3 min. We performed AFM and GIXD characterizations on this sample in each condition; the morphological image and crystal structure patterns of the sample were almost the same. In fact, the AFM images which were taken in the exact same area showed similar nucleation density, islands positions and islands heights. Moreover, the GIXD patterns clearly showed the same *fcc* crystal structure with the same (111) domain size. Therefore, we confirmed that any damage to C_{60} thin film by gold etchant is insignificant (Figure S8).

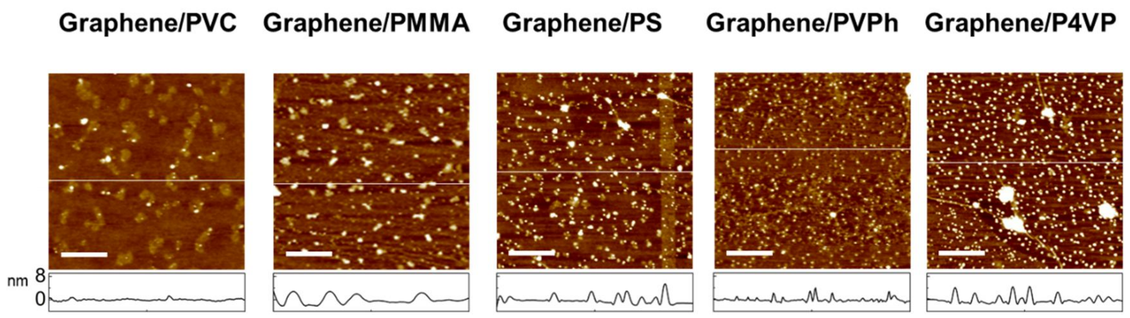

Figure S9. Morphology of 0.25 ML C_{60} thin films grown on polymer-contact doped graphene. Scale bar: 400 nm.

Morphology of C_{60} films grown on graphene. The morphology was examined using atomic-force microscopy (AFM, Bruker) in tapping mode. The height, surface coverage and nucleation density were analyzed using NanoScope Analysis software.

For the general observation, both electrical-gate doped and polymer-contact doped graphene templates were used separately for growth of C_{60} crystals. The morphology of C_{60} and the trend of results on both kinds of graphene templates were similar (Figure 4 in the main text and Figure S9); this result confirms the effects of charge transfer between graphene and C_{60}, but not the other substrate effects, on the growth behavior of C_{60}.

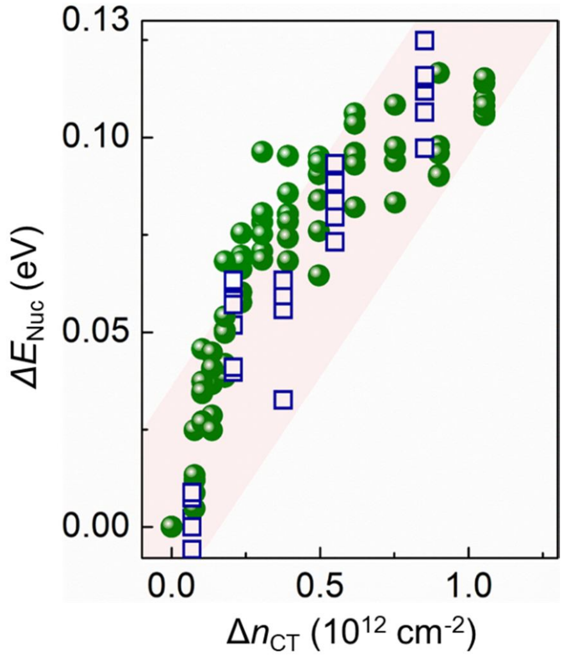

Figure S10. Plot of ΔE_{Nuc} vs. Δn_{CT} at 0.25 ML thick of C_{60} thin films grown on graphene

Effects of charge transfer on C_{60} crystal's growth. To quantify the dependence of C_{60} growth on charge transfer between the C_{60} ad-molecules and the graphene template, numerous C_{60} thin films with nominal thickness of 0.25 ML were grown on graphene templates in which E_F had been finely controlled using either electrical gating or polymer doping. The plot of the nucleation density N_i of these films against Δn_{CT} reveals the correlations between nucleation of C_{60} and the charge transfer between graphene and C_{60} (Figure 4c in the main text). Here we introduce the term ΔE_{Nuc} to represent the difference in activation energy for a C_{60} ad-molecule to nucleate between at a significant Δn_{CT} and at Δn_{CT} ~0. Clearly, Δn_{CT} determined N_i and the activation energy for C_{60} nucleation E_{Nuc} (Figure S10). E_{Nuc} can be extracted using $N_i = C \cdot \exp(E_{Nuc}/(k_B T))$ where C is a pre-exponential factor, k_B is the Boltzmann constant, and T is the substrate temperature.

At room temperature, as Δn_{CT} increased, N_i increased, so E_{Nuc} also increased, with a linear slope of 1.2×10^{-13} eV/cm^2 (Figure S10). We also directly measured E_{Nuc} by analyzing the dependence of N_i on T at different Δn_{CT}. E_{Nuc} was obtained as the slope of a plot of $\ln(N_i)$ vs.

$1/(k_BT)$ (Figure 4d in the main text). These results means that E_{Nuc} increased with a linear slope of 1.3×10^{-13} eV/cm^2 as Δn_{CT} increased (Figure 4e in the main text). The similarity of these two slopes (Figure 4e in the main text and Figure S10) confirms that the dependence of N_i on Δn_{CT} is attributable to the dependence of E_{Nuc} on Δn_{CT}.

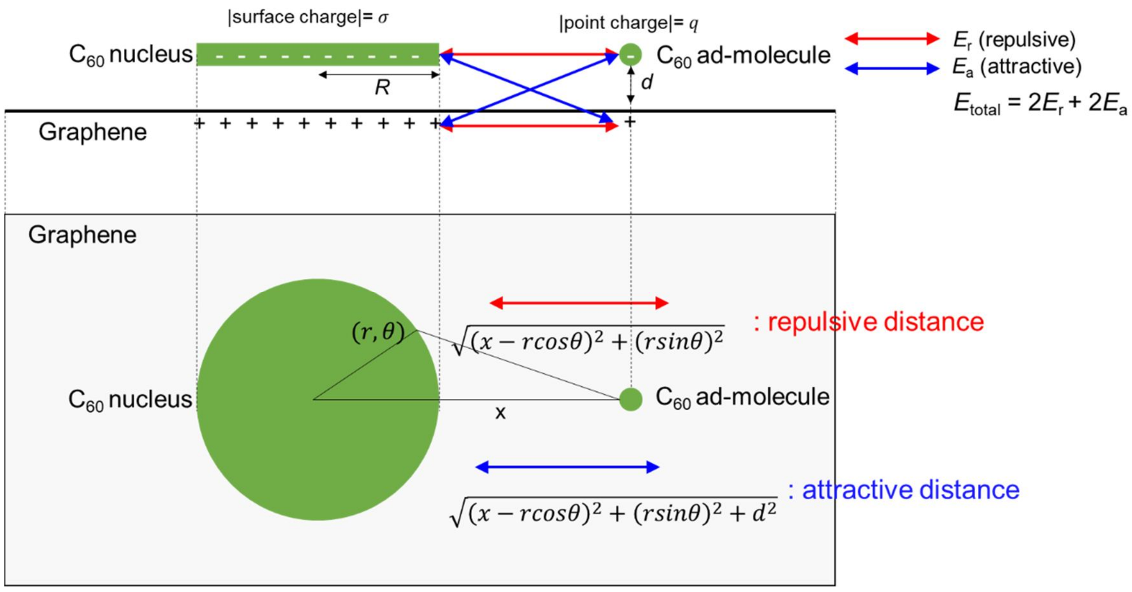

Figure S11. Schematic illustration of a C_{60} island and a C_{60} ad-molecule on graphene substrate along with calculation details.

Mechanism of C_{60} growth on graphene. When electrons in graphene are transferred to a C_{60} island or a C_{60} ad-molecule, an electrostatic repulsion develops between the island and the negatively-charged ad-molecule (Figure S11). Additional electrostatic attractions develop between negatively-charged ad-molecules or islands, and positively-charged graphene beneath them. Here, we tried to calculate the additional attachment barrier E_B' that these electrostatic interactions induce.

Assuming the C_{60} island is a circular plate, the electrostatic potential energy that corresponds to repulsion can be calculated as

$$E_r = \iint \frac{1}{4\pi\varepsilon_0} \frac{e^2 Z_{avg} \Delta n_{CT}}{\sqrt{(x-rcos\theta)^2+(rsin\theta)^2}} r dr d\theta, \qquad \text{(Equation S1)}$$

and the electrostatic potential energy that corresponds to attraction can be calculated as

$$E_a = -\iint \frac{1}{4\pi\varepsilon_0} \frac{e^2 Z_{avg} \Delta n_{CT}}{\sqrt{(x-rcos\theta)^2+(rsin\theta)^2+d^2}} r dr d\theta, \qquad \text{(Equation S2)}$$

where the parameters are related to the geometry of the island (Figure S9), and the integrations are done over the surface of the C_{60} island. Then the total electrostatic potential

energy E_{total} of the system is simply $2(E_r + E_a)$. Using dimensionless parameters $x' = x/R$, $r' = r/R$ and $d' = d/R$, E_{total} can be expressed as

$$E_{total}(x'; d, R) = \frac{e^2 Z_{avg} \Delta n_{CT}}{\varepsilon_0} f(x'; d/R), \qquad \text{(Equation S3)}$$

$$f(x'; d/R) = \left[\frac{1}{2\pi}\int_0^{2\pi}\int_0^1 \frac{1}{d'}\left(\frac{r'}{\sqrt{(x'-r'cos\theta)^2+(r'sin\theta)^2}} - \frac{r'}{\sqrt{(x'-r'cos\theta)^2+(r'sin\theta)^2+d'^2}}\right)dr'd\theta\right] \quad .$$

(Equation S4)

The Δn_{CT}-dependent attachment barrier V_B is the limit of E_{total} as x' approaches 1,

$$E_B' = \lim_{x'\to 1} E_{total} = \frac{Z_{avg} e^2 d \Delta n_{CT}}{\varepsilon} f(1; d/R). \qquad \text{(Equation S5)}$$

where $f(1; d/R) \approx 0.5$, so $E_B' \approx Z_{avg} e^2 d \Delta n_{CT}/(2\varepsilon)$.

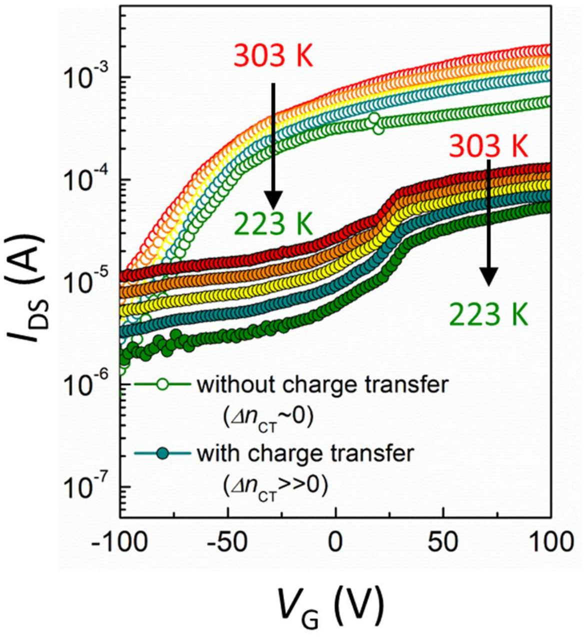

Figure S12. **Graphene-C_{60} barristors.** Temperature-dependent I_{DS}-V_G of barristors fabricated with C_{60} films grown on graphene without charge transfer (open) and with charge transfer (closed).

C_{60} field-effect transistors and graphene-C_{60} barristors. For a C_{60} field-effect transistor (C_{60}-FET), C_{60} film with 150-nm thickness was firstly grown on gate-biased graphene on SiO_2/Si substrate. Then part of this C_{60} layer (~50 nm) was transferred onto octadecyltrichlorosilane (ODTS)-treated 300-nm SiO_2/*p*-Si substrates by PDMS stamping. The method to treat SiO_2/Si substrate with ODTS has been described elsewhere.[4]

The key of this transfer method is that PDMS contacts only the C_{60} film and not the bottom graphene, so that the PDMS piece could lift ~50-nm-thick C_{60} film up and place it on the target substrate. The rest of the C_{60} film remained on graphene on the initial substrate. The whole transfer process was conducted in a glove box under inert N_2 atmosphere to avoid oxidation of C_{60}. Although perfect transfer large-area C_{60} films by the stamping method was not easy, we could efficiently obtain small (~1 cm^2) crack-free C_{60} films. Then we fabricated channels (1000 μm × 50 μm) by depositing 100-nm-thick Al source and drain electrodes on these intact transferred C_{60} films.

The measurements were conducted using a Keithley 2400 in vacuum (10^{-6} Torr) to avoid oxidation.

The electron mobility μ_e of the C_{60}-OFET was extracted in the saturation region (V_{DS} = 100 V) by using the equation:

$$I_{DS} = \frac{WC_i}{2L}\mu_e(V_G - V_T)^2, \qquad \text{(equation S6)}$$

where I_{DS} is the drain current, C_i is the capacitance of the oxide, V_G is the gate voltage and V_T is the threshold voltage.

To fabricate a barristor, a 150-nm-thick layer of C_{60} was deposited on gate-biased graphene on SiO_2/Si substrate through a shadow mask. Then 100-nm Au was deposited onto graphene as a source electrode, and 100-nm Al was deposited on the C_{60} layer as a drain electrode using shadow masks. The devices were characterized inside the vacuum probe station. To avoid affecting the delicate morphology of C_{60} film, the measurement temperature was varied from 223 to 303 K (Figure S12).

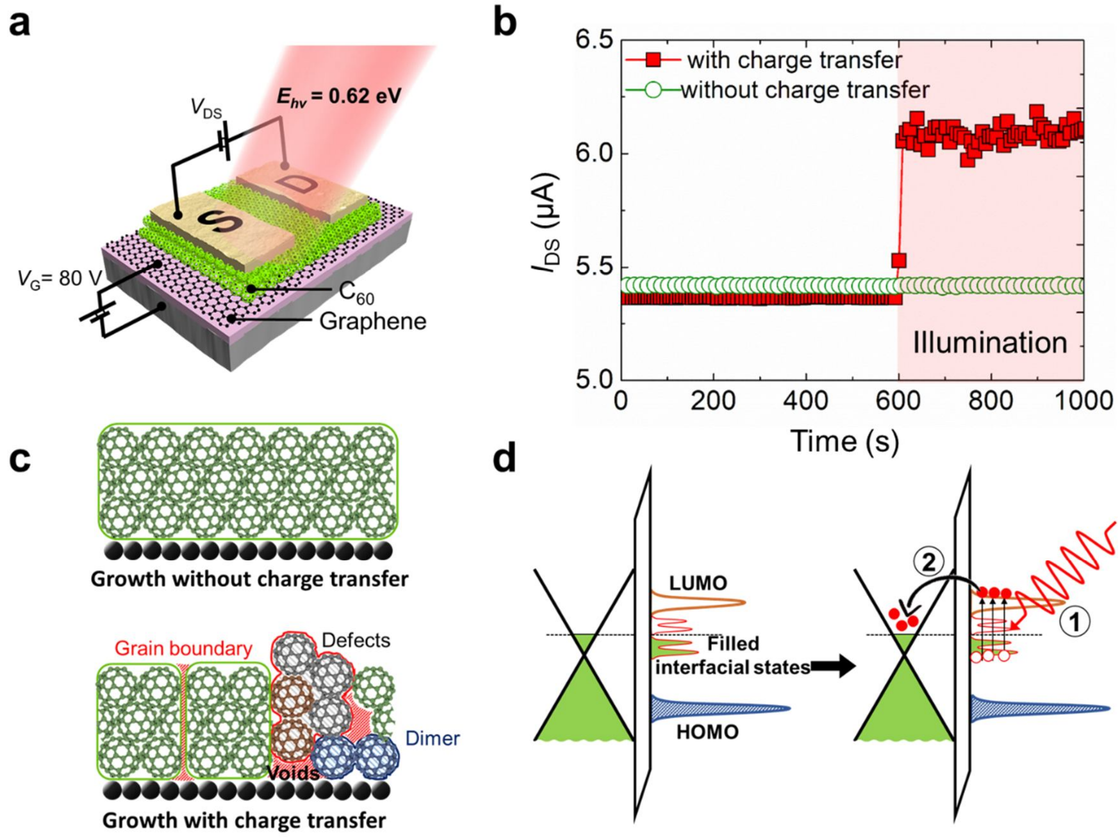

Figure S13. **De-trapping effects of low-energy wavelength on C_{60} thin films grown on graphene with and without charge-transfer effect**. **a,** Schematic of the device. **b,** Current vs. measuring time including illumination period. **c,** Schematic of the interface between graphene and C_{60} where C_{60} film was grown without and with charge transfer. **d,** Proposed mechanism of the presence of photocurrent, (1) photo-absorption and charge de-trapping and (2) transferring de-trapped electrons back to graphene.

Interfacial states of C_{60} thin film confirmed by photocurrent measurements. To measure the charge de-trapping effects, we fabricated graphene/C_{60} transistors (Figure S13a). The thickness of C_{60} layers was 20 nm, grown with or without charge-transfer effect. The channel size was 10 μm × 1000 μm. The measurement was performed in a vacuum chamber (10^{-6} Torr) equipped with an optical fiber connecting to a monochromator. The beam energy was 0.62 eV, V_G was 80 V, V_{DS} was 0.05 V.

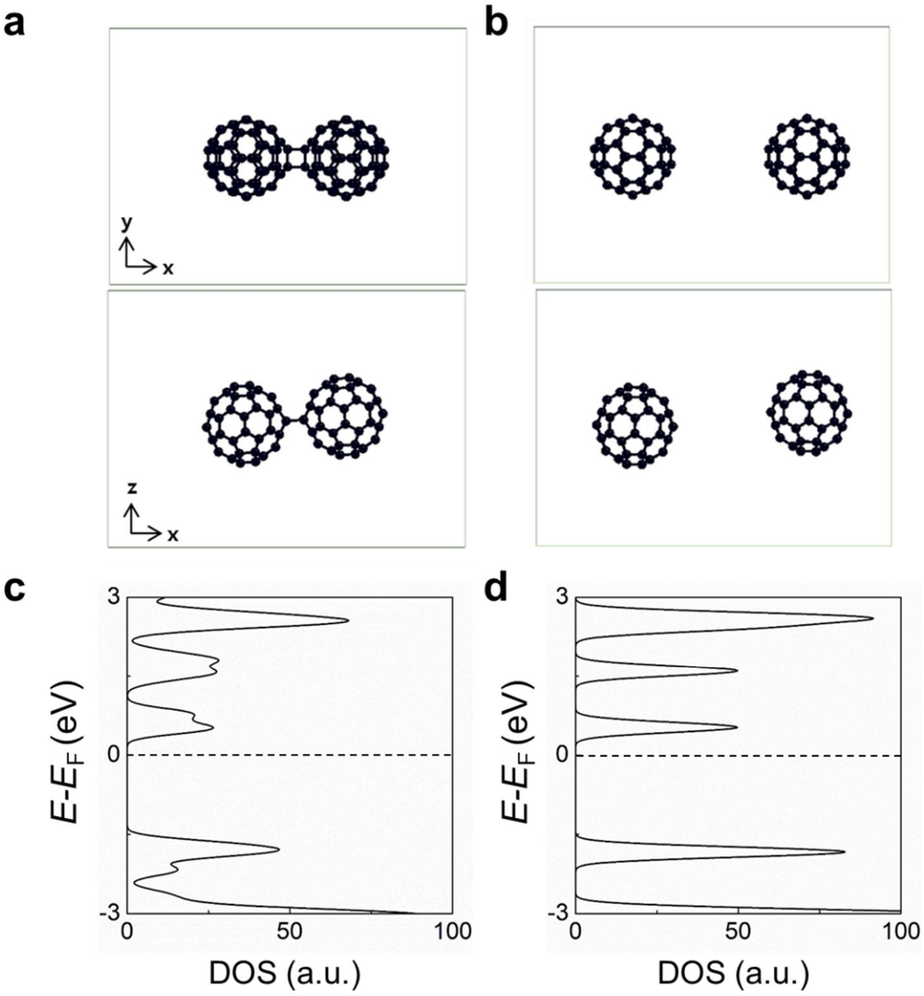

Figure S14. DFT calculation for structures of C_{60} dimer, and corresponding DOS, respectively. Unit cell of **a,** $(C_{60})_2$, **b,** $2C_{60}$ after structure relaxation (Top: x-y plan view, Bottom: x-z plan view). DOS of C_{60} dimers with c, structure of $(C_{60})_2$ and **d,** $2C_{60}$.

Density function state (DFT) calculations of C_{60} molecules. Initial single C_{60} structure was adopted from the *fcc* crystal C_{60}. Then 16-Å vacuum slaps in all Cartesian coordinates were set for the C_{60} dimer before relaxation to avoid interaction of the periodic lattice. To determine the configuration of bonded C_{60} dimer, each C_{60} was initially positioned with a distance of 3.3 Å and fully relaxed to its lowest energy. To compare the crystal and electronic structure of C_{60} dimer before and after bonding, we also calculated the structure of isolated

C_{60} dimer; a 9 Å distance was set for isolated C_{60}. We abbreviate the bonded dimer as $(C_{60})_2$ and the isolated 2 C_{60} as $2(C_{60})$.

The relaxed structure and the corresponding density of states (DOS) were calculated using Quantum Espresso 6.3.0 ver.,[5] and Plain Augment Waves, Optimized Norm-Conserving Vanderbilt pseudopotentials from the PAL library.7.[6] The cut-off energies and k-grid for relaxed structure and DOS were set to ecutwfc = 40 Ry for Gamma point and 60 Ry for 3 × 3 × 1. The convergence threshold on force was set to 1.0D-6 for calculation of optimized structures. A small scissors-operator of 0.6 eV was applied.

References

[1] N. N. Nguyen, H. C. Lee, B. Kang, M. Jo, K. Cho, *Nano Lett.* **2019**, *19*, 1758.

[2] N. N. Nguyen, S. B. Jo, S. K. Lee, D. H. Sin, B. Kang, H. H. Kim, H. Lee, K. Cho, *Nano Lett.* **2015**, *15*, 2474.

[3] a) F. Xia, V. Perebeinos, Y.-m. Lin, Y. Wu, P. Avouris, *Nature Nanotechnol.***2011**, *6*, 179; b) F. Giubileo, A. Di Bartolomeo, *Prog. Surf. Sci.* **2017**, *92*, 143.

[4] H. S. Lee, D. H. Kim, J. H. Cho, M. Hwang, Y. Jang, K. Cho, *J. Am. Chem. Soc.* **2008**, *130*, 10556.

[5] a) P. Giannozzi, O. Andreussi, T. Brumme, O. Bunau, M. Buongiorno Nardelli, M. Calandra, R. Car, C. Cavazzoni, D. Ceresoli, M. Cococcioni, N. Colonna, I. Carnimeo, A. Dal Corso, S. de Gironcoli, P. Delugas, R. A. DiStasio, A. Ferretti, A. Floris, G. Fratesi, G. Fugallo, R. Gebauer, U. Gerstmann, F. Giustino, T. Gorni, J. Jia, M. Kawamura, H. Y. Ko, A. Kokalj, E. Küçükbenli, M. Lazzeri, M. Marsili, N. Marzari, F. Mauri, N. L. Nguyen, H. V. Nguyen, A. Otero-de-la-Roza, L. Paulatto, S. Poncé, D. Rocca, R. Sabatini, B. Santra, M. Schlipf, A. P. Seitsonen, A. Smogunov, I. Timrov, T. Thonhauser, P. Umari, N. Vast, X. Wu, S. Baroni, *J. Phys. Condens. Matter* **2017**, *29*, 465901; b) P. Giannozzi, S. Baroni, N. Bonini, M. Calandra, R. Car, C. Cavazzoni, D. Ceresoli, G. L. Chiarotti, M. Cococcioni, I. Dabo, A. Dal Corso, S. de Gironcoli, S. Fabris, G. Fratesi, R. Gebauer, U. Gerstmann, C. Gougoussis, A. Kokalj, M. Lazzeri, L. Martin-Samos, N. Marzari, F. Mauri, R. Mazzarello, S. Paolini, A. Pasquarello, L. Paulatto, C. Sbraccia, S. Scandolo, G. Sclauzero, A. P. Seitsonen, A. Smogunov, P. Umari, R. M. Wentzcovitch, *J. Phys. Condens. Matter* **2009**, *21*, 395502.

[6] A. Dal Corso, *Comput. Mater. Sci.* **2014**, *95*, 337.
